# Individual fixation tendencies in person viewing generalize from images to videos

**DOI:** 10.1177/20416695221128844

**Published:** 2022-11-04

**Authors:** Maximilian D. Broda, Benjamin de Haas

**Affiliations:** 9175 Department of Experimental Psychology, Justus Liebig University Giessen, Germany; Center for Mind, Brain and Behavior (CMBB), University of Marburg and Justus Liebig University Giessen, Germany

**Keywords:** eye movements, individual differences, face perception, objects and features, scene perception

## Abstract

Fixation behavior toward persons in static scenes varies considerably between individuals. However, it is unclear whether these differences generalize to dynamic stimuli. Here, we examined individual differences in the distribution of gaze across seven person features (i.e. body and face parts) in static and dynamic scenes. Forty-four participants freely viewed 700 complex static scenes followed by eight director-cut videos (28,925 frames). We determined the presence of person features using hand-delineated pixel masks (images) and Deep Neural Networks (videos). Results replicated highly consistent individual differences in fixation tendencies for all person features in static scenes and revealed that these tendencies generalize to videos. Individual fixation behavior for both, images and videos, fell into two anticorrelated clusters representing the tendency to fixate faces versus bodies. These results corroborate a low-dimensional space for individual gaze biases toward persons and show they generalize from images to videos.

## Introduction

Where humans look in scenes can be predicted by the presence of objects with semantic features (de Haas et al., 2019; Guy et al., 2019; Linka & de Haas, 2020; Xu et al., 2014). Human gaze is particularly attracted by social stimuli, both in static (End & Gamer, 2017) and dynamic scenes (Rubo & Gamer, 2018; Smith & Mital, 2013). Faces attract early fixations in static scenes (Cerf et al., 2009) and elicit saccades faster than other semantic objects in saccadic choice tasks (Broda et al., 2022; Crouzet et al., 2010). Faces are also highly salient in the context of videos (Foulsham et al., 2010; Klin et al., 2002). At the same time, the tendency to fixate faces and eyes is modulated by robust individual differences, (Constantino et al., 2017; Guy et al., 2019; Peterson & Eckstein, 2013) and these fixation tendencies correlate with face recognition and memory performance (de Haas et al., 2019; Linka, Broda, et al., 2022). For special populations such as humans with autism spectrum disorder, the avoidance of faces and eyes in particular (Tanaka & Sung, 2016) has been linked to a general reduction of social attention (Riby & Hancock, 2008).

Previous work suggests that individual differences in saccade dynamics are a function of the observer and largely independent of the type of stimulus (Andrews & Coppola, 1999). However, whether content-dependent fixation biases generalize across different stimulus modalities as well is largely unclear. We recently found that the individual tendencies to fixate faces and eyes in static scenes are highly correlated with each other, but anticorrelated with the tendency to fixate other body parts (Broda & de Haas, 2022). Whether such individual tendencies and their intercorrelations generalize from static to dynamic scenes is an important question for their theoretical interpretation. If these gaze biases are related to fundamental biological differences, like the individual functional layout of the ventral stream (Broda & de Haas, 2022; de Haas et al., 2019), they should generalize from image viewing to videos. While individually preferred saccadic landing points generalize from faces on a screen to real-world interactions (Peterson et al., 2016), fixations toward faces and their features in videos can be modulated by viewing angle and sound, particularly speech (Foulsham et al., 2010; Vo et al., 2012), rendering the stimulus specificity of these biases unclear. In general, individual differences in gaze behavior seem attenuated for director-cut videos due to motion onsets and cuts (Dorr et al., 2010; Mital et al., 2011; Schütz et al., 2011). Here, we tested the strong hypothesis that observer-specific preferences in person looking generalize between static scenes and director-cut videos nonetheless.

## Methods

### Subjects

Forty-four participants completed the experiment (age: *M*  =  24 years; *SD*  =  5; 34 females), but data from one participant was excluded due to excessive data loss during the video viewing. All participants had normal or corrected to normal vision. The study was approved by the local ethics committee at Justus Liebig University Giessen and conducted in accordance with the declaration of Helsinki except preregistration. All participants gave informed consent before the experiment and were compensated with 8€/h or course credits.

### Images and Pixel Masks

The Object and Semantic Images and Eye-tracking (OSIE) set is publicly available and consists of 700 static natural scenes (https://www-users.cse.umn.edu/∼qzhao/predicting.html) with hand-drawn pixel masks and categorical semantic labels for 5551 objects (Xu et al., 2014). For our analysis, we used the OSIE Person stimulus set that consists of 6365 hand-delineated pixel masks and labels for different person features (Broda & de Haas, 2022).

### Videos and Deep Neural Networks

Participants viewed eight different director-cut video clips downloaded from YouTube, which included social content and were similar to videos used in a previous study (Parkinson et al., 2018; Table S1). All videos were presented in German and at a frame rate of 25 Hz. We used publicly available Deep Neural Networks (DNNs) for feature labeling, specifically a Cross-Domain Complementary Learning (CDCL) DNN to segment different body parts in all frames of all videos (Lin et al., 2021). Additionally, we used the RetinaFace DNN for annotations of eyes and mouths (Deng et al., 2020).

### Apparatus

Participants placed their head in a chin and forehead rest and viewed stimuli at a distance of 55 cm at 34.3  ×  25.7 degrees visual angle (images) or 36.6  ×  20.6 degrees visual angle (videos). The experiment was controlled and data was analyzed via Psychtoolbox (Kleiner et al., 2007) and MATLAB (MathWorks, Natick, MA). Gaze data were acquired using an EyeLink 1000 Plus eye tracker (SR Research, Ottawa, Canada) at a frequency of 1 kHz.

### Procedure

Participants freely viewed all 700 images which were presented in the same order for all. Images were split into seven blocks of 100 each. A nine-point (re)calibration was done before the start of each block if participants left the chin rest (validation error: *M*  =  0.37 degrees visual angle (dva), *SD*  =  0.10), so participants could pause the experiment and leave the chin and forehead rest in between blocks. Each trial started with a central fixation disk and participants could trigger the trial start via a button press. Image presentation lasted two seconds which is enough to detect individual differences (Linka & de Haas, 2020). Afterwards, participants viewed the eight videos in the order listed in Table S1. Again, they were allowed to pause the experiment between videos and underwent recalibration before each video (validation error: *M*  =  0.38 dva, *SD*  =  0.09; note that all validation error statistics were based on data from 41 participants as calibration data was lost for two). Participants started each video with a central fixation on a disk, followed by a button press. The complete experiment lasted between 90 and 120 min.

### Analysis

For the present study, we analyzed fixations that fell on the following features: arms, hands, torsi, legs, heads, eyes, and mouths (see Table S2 for different feature sizes). Fixations were labeled if they landed on a feature or were within a distance of 0.5 degrees visual angle from the respective feature mask in images or video frames. Fixations labeled for more than one feature were excluded from further analyses unless the respective features are naturally overlapping (e.g., head and inner face features).

#### Images

We restricted all analyses to the images that contained person depictions (469). All fixations earlier than 100 ms after image onset (onset fixations) and shorter than 100 ms in duration were excluded from further analyses. First fixations were defined as the first fixation in each trial (excluding onset fixations). *Proportion of First Fixations* was defined by adding the number of cases in which the first fixation after image onset landed on a given feature, divided by the number of cases the first fixation landed on any person feature. *Proportion of Dwell Time* was defined across all fixations that landed on person features. We calculated both indices for each observer and feature, separately for odd, even, and all trials. We assessed the consistency of individual differences in fixation behavior by correlating individual fixation proportions across odd and even trials and computed zero-order correlations between individual fixation proportions for all features, separately for first fixations and dwell times. The resulting correlations were then subjected to multidimensional scaling to project them onto a two-dimensional plane and visualize their structure.

Additionally, we calculated the proportion of all fixations (not just those landing on person features), which landed on the body and head, separately for first fixations and dwell time. Body features consisted of arms, hands, torsi, and legs if visible for a depicted person.

Finally, control analyses were restricted to the 30%, 20%, and 10% largest person masks to control for possible calibration artifacts.

#### Videos

We first manually excluded frames with no visible body features to reduce the number of false positive feature annotations by the DNNs. We then checked 31,955 gaze samples across 800 frames (five randomly chosen packages of 20 consecutive frames for each video), revealing that <8% of them fell on erroneous mask labels.

*Proportion of Video Fixations* was defined by adding the number of frames in which participants’ gaze position landed on a given feature, divided by the number of frames their gaze position landed on any person feature. To determine the consistency of individual fixation tendencies for videos, we calculated the median split-half correlation for every feature across all possible splits of the eight videos into two groups of four (70 combinations). As for images, we also computed zero-order correlation matrices between fixation proportions and subjected them to multidimensional scaling. Additionally, we calculated the *Proportion of Video Fixations* for the features head and body across gaze positions of all frames. The body feature combined the masks for arms, hands, torsi, and legs for a given depicted person.

#### Generalization Between Images and Videos

To test, whether fixation behavior generalizes from images to videos, we correlated the *Proportion of Video Fixations* with the *Proportion of First Fixations* and *Proportion of Dwell Time*, respectively. This was done across all trials and for all features, separately. Additionally, we tested to which degree the covariance patterns of fixation tendencies generalized from images to videos by correlating the respective (Fisher *z*-transformed, off-diagonal) zero-order correlation matrices with each other. Finally, we tested whether the tendency to fixate any person feature (across all fixations) generalizes from images to videos.

All correlations were calculated using Pearson's correlation coefficient. Split-half consistencies were additionally corrected for attenuation using the Spearman–Brown formula. Significance was determined at a family-wise error (FWE) rate of α  =  .05 using the Holm–Bonferroni method to correct for multiple testing (reported *p*-values are uncorrected, but only marked as significant if they survived FWE correction).

## Results

### Images: Split-Half Consistencies and Covariance Patterns

**Figure 1.**
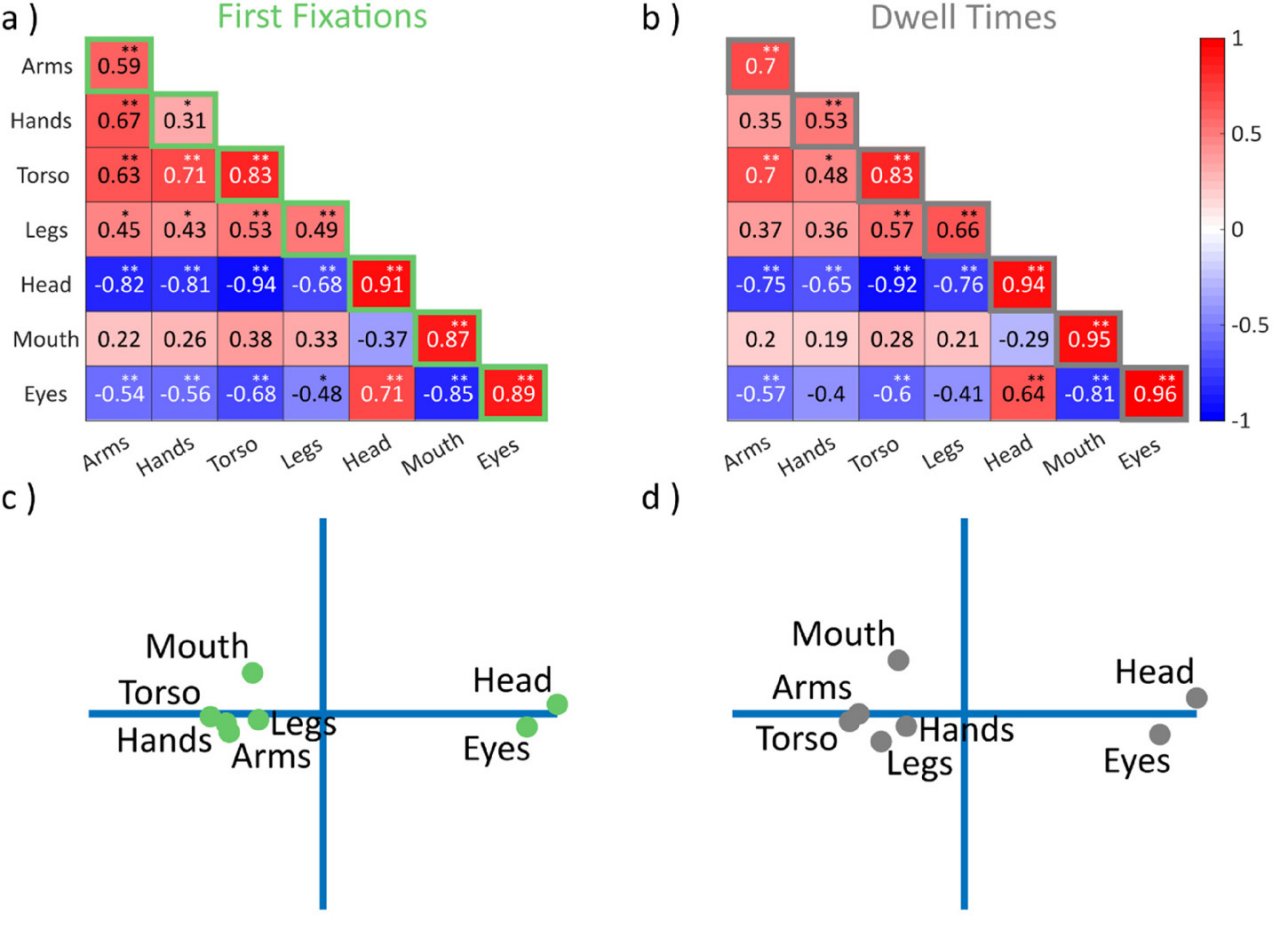
Split-half consistencies and interfeature correlations (images). Correlation matrices in (a, b) show the covariance between fixation tendencies in off-diagonal cells and their split-half consistencies on the diagonal for (a) proportions of first fixations and (b) proportional dwell times. Negative to positive correlations are indicated by color and saturation, as shown on the color bar to the right. Asterisks indicate statistical significance (Holm–Bonferroni corrected for multiple testing) **p < .001, *p < .05. (c, d) show the corresponding two-dimensional projections derived with multidimensional scaling.

Split-half correlations revealed medium to strong consistencies ranging from *r*(41)  =  .31, *p*  =  .043 (hands) to *r*(41)  =  .91, *p* < .001 (head) for proportions of first fixations and between *r*(41)  =  .53, *p* < .001 (hands) and *r*(41)  =  .96, *p* < .001 (eyes) for proportional dwell times (diagonal values in Figure 1a and b; Figure S1a, c, and e for example images). Spearman–Brown corrected split-half correlations indicated strong consistencies for all features (First: all *r*(41) > .45; Dwell: all *r*(41) ≥ .7; Table S3).

**Figure 2.**
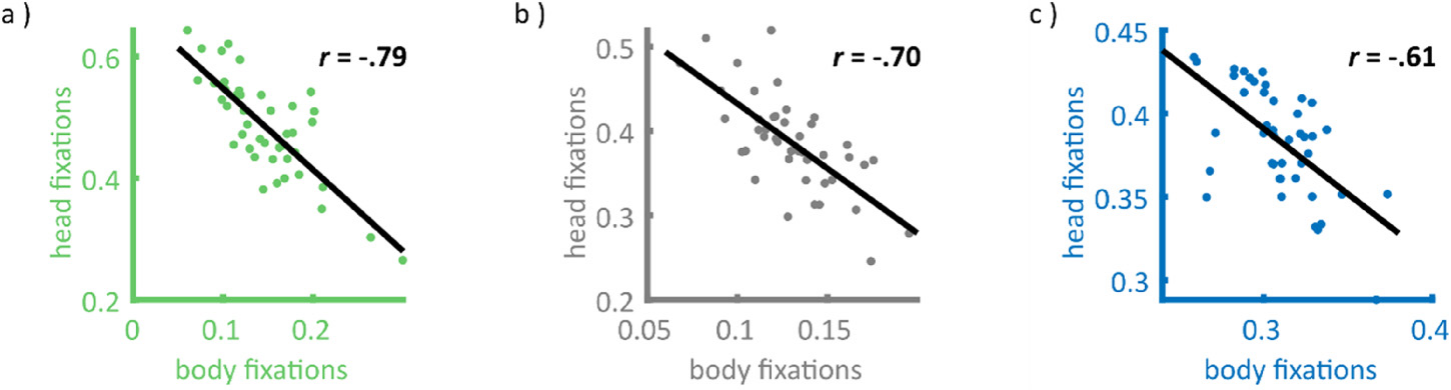
Head versus body fixations across all fixations. The figure shows the scatter plots for the negative correlation between the features head and body across all fixations (not just those landing on persons). Each scatter point represents the proportion of fixations of a single observer, which landed on the respective feature for (a) proportions of first fixations, (b) proportional dwell time, and (c) the proportion of video gaze samples. The least-square lines are shown in black and corresponding correlation values are written in the top right of each plot.

Correlating fixation tendencies for different features with each other revealed significant correlations between all body features outside the head for proportions of first fixations (all *r*(41) > .4) and between torsi and all other body features for proportional dwell times (all *r*(41) > .45). Additionally, head and eyes were significantly correlated with each other (First: *r*(41)  =  .71, *p* < .001; Dwell: *r*(41)  =  .64, *p* < .001). Mouth fixations showed no significant positive correlation with any other feature but were negatively associated with eye fixations (First: *r*(41)  =  −.85, *p* < .001; Dwell: *r*(41)  =  −.81, *p* < .001; Figure 1a and b). Multidimensional scaling highlighted different clusters for body and face features and showed that mouths fell outside the face and into the body cluster (Figure 1c and d). Similarly, when considering all fixations (not just person-directed fixations) the tendencies to fixate bodies and heads showed strong negative correlations for first fixations, *r*(41)  =  −.79, *p* < .001, and dwell time, *r*(41)  =   .70, *p* < .001 (Figure 2a and b).

This covariance pattern proved highly stable in control analyses restricted to features from the 30%, 20%, or 10% largest person masks (all resulting covariance patterns *r* ≥ .9 with the original analysis).

### Videos: Split-Half Consistencies and Covariance Patterns

**Figure 3.**
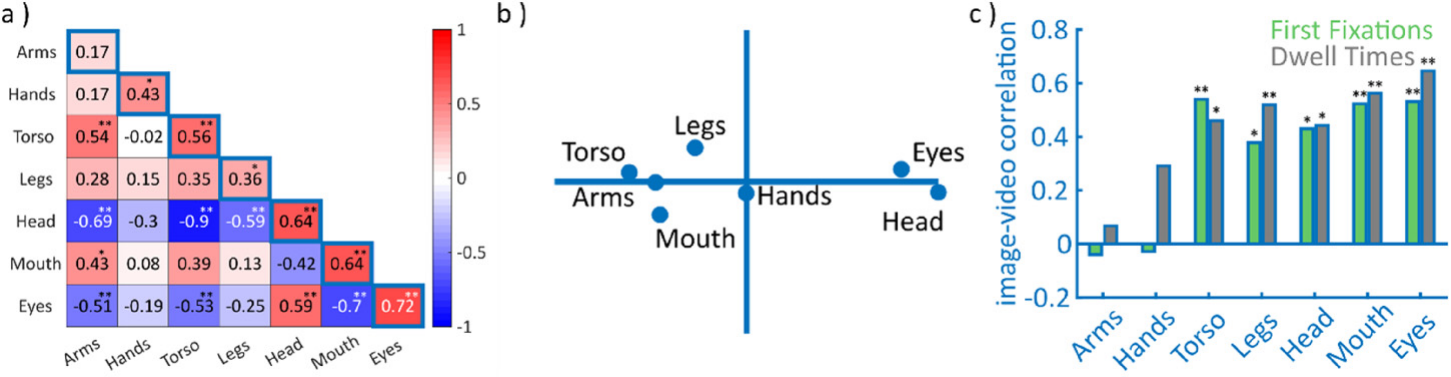
Split-half consistencies and interfeature correlations (videos). The correlation matrix in (a) shows the covariance between gaze tendencies in off-diagonal cells and their split-half consistencies on the diagonal for proportions of video fixations. (b) shows the corresponding two-dimensional projection derived with multidimensional scaling. (c) shows the correlations between the proportion of video gaze samples and the proportion of first fixation (green) or proportional dwell time (grey) in images, respectively, for all features. Asterisks indicate statistical significance (Holm–Bonferroni corrected for multiple testing) **p < .001, *p < .05.

Median split-half correlations for gaze tendencies during video watching revealed consistencies that ranged from *r*(41)  =  .17, *p*  =  .266 (arms) to *r*(41)  =  .72, *p* < .001 (eyes; Figure 3a; Figure S1b, d, and f for example frames). Spearman–Brown split-half correlations indicated medium to strong consistencies ranging from *r*(41)  =  .30 (arms) to *r*(41)  =  .84 (eyes; Table S3). The tendencies to fixate body features (outside the head) were not significantly correlated with each other apart from torso and arms (*r*(41)  =  .54, *p* < .001). The tendencies to fixate heads and eyes were significantly correlated (*r*(41)  =  .59, *p* < .001) while showing negative correlations with other body features. Similar to the results for images, mouth fixations were independent of face fixations, only showing a moderate correlation with the tendency to fixate arms (*r*(41)  =  .43, *p*  =  .004) and a negative correlation with the tendency to fixate eyes (*r*(41)  =  −.70, *p* < .001), as reflected in separate multidimensional scaling clusters for head and eyes on the one hand and all other body parts on the other hand (Figure 3b). Again, proportions of the body and head fixations across all frames were also negatively correlated, *r*(41)  =  −.61, *p* < .001 (Figure 2c).

### Consistencies Across Images and Videos

The covariance patterns of fixation tendencies were highly similar across images and videos, as confirmed by their significant correlation (First: *r*(41)  =  .91, *p* < .001; Dwell: *r*(41)  =  .95, *p* < .001). Similarly, the tendency to direct a person fixation toward a given feature was significantly correlated across images and videos for all features but arms and hands (Figure 3c; see Figure S2a and b for complete covariance patterns). Finally, the overall tendency to fixate any person feature showed no significant correlation (both *r* ≤ .2, *p* > .19; Figure S2c).

## Discussion

The current study tested whether individual gaze biases for person features generalize from static scenes to director-cut videos. We segmented seven person features (arms, hands, torso, legs, head, mouth, and eyes) of depicted persons using hand-delineated pixel masks for images (Broda & de Haas, 2022) and DNNs for videos (Deng et al., 2020; Lin et al., 2021) to quantify the individual distribution of gaze across these features.

Our results replicate previous findings showing medium to strong consistencies for individual fixation tendencies toward person features in static scenes (Broda & de Haas, 2022; de Haas et al., 2019; Guy et al., 2019; Linka, Broda, et al., 2022; Linka & de Haas, 2020; Peterson et al., 2016). Specifically, we replicate two distinct clusters for face (head & eyes) and body features, with the tendency to fixate mouths falling closer to the body cluster and a strong negative correlation for the general tendency to fixate heads versus bodies (Broda & de Haas, 2022). Crucially, observers also showed consistent fixation tendencies for person features in videos and the resulting covariance pattern was highly similar to that seen for image viewing. While the consistency of fixation tendencies for videos generally was lower than for static scenes, the Spearman–Brown corrected estimates for the consistency across eight video clips were at least moderate for all features (minimum *r*  =  .30). Most importantly, individual fixation tendencies proved consistent across images and videos.

Several factors may contribute to the somewhat lower consistency of individual fixation tendencies in videos compared to images. Inspecting the DNN-derived labels of body parts in videos confirmed sufficient quality, but also revealed some errors rendering them less precise than the hand-delineated pixel masks used for image annotations. Furthermore, director-cut videos have been shown to attenuate interindividual variance in gaze, due to a strong center bias (Goldstein et al., 2007; Schütz et al., 2011) and the salience of motion onsets (Dorr et al., 2010; Mital et al., 2011).

The finding that individual biases in the way we fixate persons generalize from static scenes to videos is compatible with the hypothesis that they reflect stable observer traits. Previous research has shown that individual fixation biases in face looking generalize from image viewing to real-world settings (Peterson et al., 2016). However, other research has found that the real-world potential for social interaction can modulate individual gaze behavior (Rubo et al., 2020). Future research will have to determine to which degree the individual fixation tendencies we found here generalize beyond images and videos on a screen.

The stability of these biases can also inform hypotheses regarding the underlying mechanisms and potential consequences. The tendency to fixate heads and faces is correlated with the tendency to fixate eyes—but not mouths. This appears in line with the proposal that eye avoidance may reflect and exacerbate social challenges (Tanaka & Sung, 2016). The anticorrelation between the tendency to fixate heads and bodies suggests an antagonistic competition between these person features. Small initial tendencies may self-reinforce by tipping the individual visual diet toward heads or bodies early in development, increasing the salience of one at the expense of the other. This would be in line with work in macaques, finding that face-deprived monkeys failed to develop normal face salience, but instead showed an increased tendency to fixate hands. These face-deprived macaques showed smaller activation in cortical regions dedicated to faces but larger neuronal activation for hands (Arcaro et al., 2017). Developmental studies suggest that similar push–pull mechanisms may play out in human ventral cortex (Nordt et al., 2021) and gaze (Broda & de Haas, 2022; Linka, Sensoy, et al., 2022). Our results confirm a crucial prediction of this hypothesis: individual differences in the way we distribute fixations across persons should generalize beyond static scenes.

Interestingly, this cross-domain consistency in the individual distribution of person fixations across features was not matched by a similar consistency in the tendency to fixate people overall. We hypothesize that this is due to the content of the videos and a resulting ceiling effect. Our videos maximized person viewing, which resulted in little interindividual variance in the overall (high) proportion of gaze samples falling on persons (Figure S1c). Future studies could specifically target overall person saliency and use videos which simultaneously present people and other potentially salient features such as dynamic text or moving vehicles in parallel.

Taken together, our results show that individual differences in the way we look at people in scenes generalize from static to dynamic scenes, even for director-cut videos (Dorr et al., 2010; Mital et al., 2011). This is in line with the hypothesis that they reflect robust traits of the individual visual brain.

## Supplemental Material

sj-docx-1-ipe-10.1177_20416695221128844 - Supplemental material for Individual fixation tendencies in person viewing generalize from images to videos Click here for additional data file. Supplemental material, sj-docx-1-ipe-10.1177_20416695221128844 for Individual fixation tendencies in person viewing generalize from images to videos by Maximilian D. Broda and Benjamin de Haas in i-Perception
