# Supplementary material for: Individual fixation tendencies in person viewing generalize from images to videos
Source: Iperception. 2022 Nov 4;13(6):20416695221128844. doi: 10.1177/20416695221128844 (PMC9638695; doi:10.1177/20416695221128844)
Supplement: sj-docx-1-ipe-10.1177_20416695221128844 - Supplemental material for Individual fixation tendencies in person viewing generalize from images to videos [file sj-docx-1-ipe-10.1177_20416695221128844.docx]

**Supplemental Material**

Table S1 Title, duration and description of selected videos.

| *Video Title* | *Duration (min:s)* | *Description* |
| --- | --- | --- |
| *Faber Alles Gute* | 4:08 | Music |
| *Fastfood* | 2:15 | Food |
| *Faultiere* | 2:56 | Animals |
| *Fußball* | 1:24 | Sports |
| *Hochzeit* | 1:33 | Wedding |
| *Pannenshow* | 1:48 | Comedy |
| *Satirenachrichten* | 2:33 | Comedy |
| *Raumstation* | 2:18 | Space Travel |

Table S2 Mean and median image feature areas and vertical feature sizes.

|  | *Total Feature Area [dva^2^]* | | *Vertical Feature Size [dva]* | | |
| --- | --- | --- | --- | --- | --- |
| *Feature* | *Mean (SD)* | *Median (25^th^, 75^th^ quantile)* | | *Mean (SD)* | *Median (25^th^, 75^th^ quantile)* |
| *Arms* | 19.29 (24.20) | 10.04 (3.93, 25.27) | | 6.07 (3.85) | 5.36 (3.13, 8.37) |
| *Hands* | 6.47 (13.23) | 2.54 (0.95, 6.80) | | 2.87 (2.39) | 2.23 (1.24, 3.73) |
| *Torso* | 27.52 (31.88) | 15.46 (5.90, 36.37) | | 6.82 (4.43) | 5.92 (3.52, 9.35) |
| *Legs* | 21.48 (18.87) | 15.76 (6.70, 32.58) | | 6.35 (3.45) | 6.13 (3.61, 8.67) |
| *Head* | 18.72 (30.24) | 6.86 (1.14, 23.10) | | 4.45 (3.82) | 3.52 (1.37, 6.48) |
| *Mouth* | 0.48 (0.64) | 0.25 (0.11, 0.60) | | 0.56 (0.38) | 0.47 (0.30, 0.73) |
| *Eyes* | 0.43 (0.56) | 0.24 (0.10, 0.53) | | 0.53 (0.36) | 0.43 (0.26, 0.69) |

Table S3 Spearman-Brown split-half consistencies for proportions of first fixations, proportional dwell times and proportions of video gaze samples.

|  | *Arms* | *Hands* | *Torso* | *Legs* | *Head* | *Mouth* | *Eyes* |
| --- | --- | --- | --- | --- | --- | --- | --- |
| *First Fixations* | 0.74 | 0.47 | 0.91 | 0.65 | 0.96 | 0.93 | 0.94 |
| *Dwell Times* | 0.83 | 0.70 | 0.91 | 0.79 | 0.97 | 0.97 | 0.98 |
| *Video Fixations* | 0.30 | 0.61 | 0.72 | 0.53 | 0.78 | 0.78 | 0.84 |


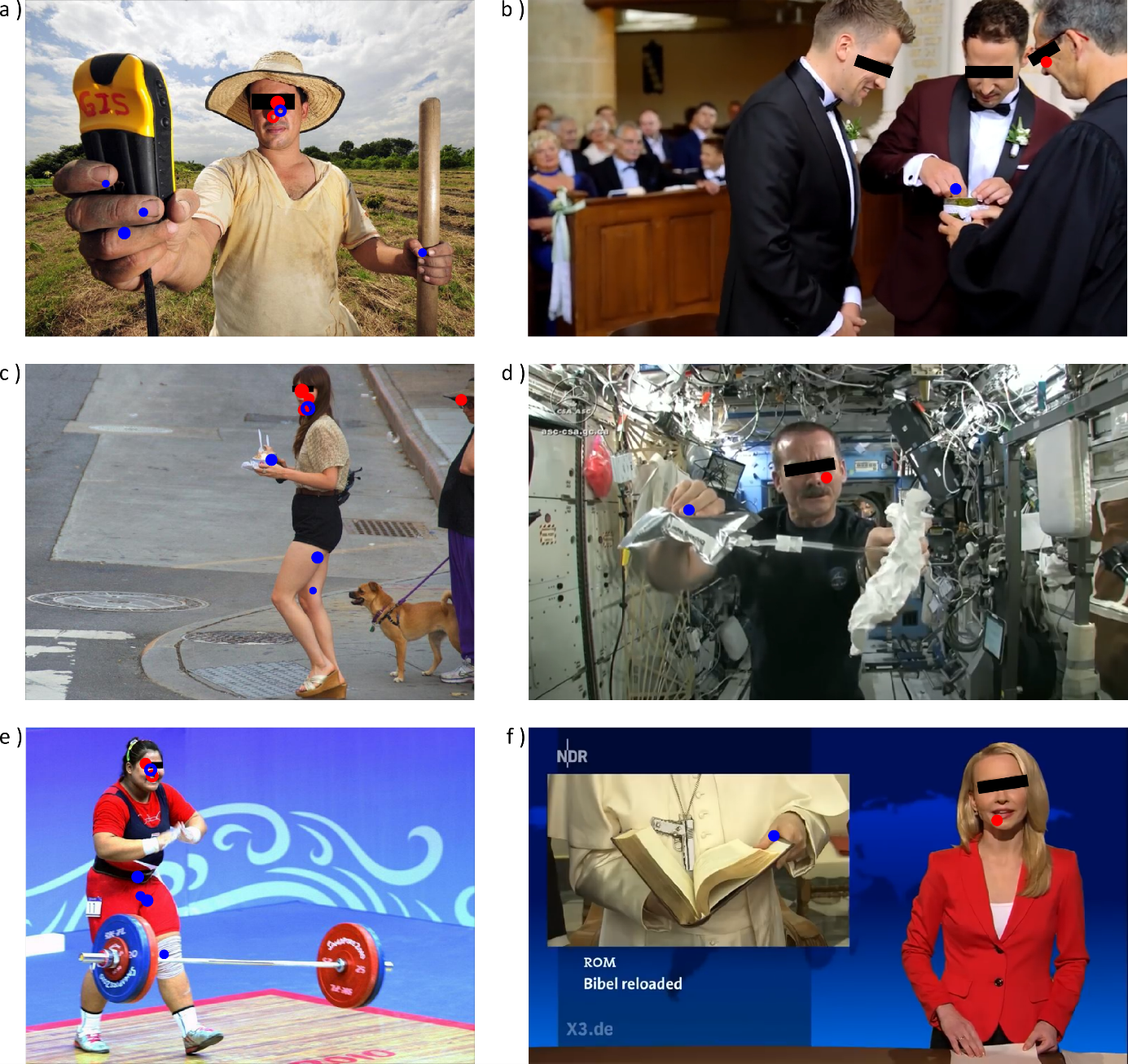


Figure S1 Example images and frames

*Panels (a - f) show example stimuli with overlaid gaze positions from two representative participants in blue (stronger body preference) and red (stronger face preference). Panels (a, c, e) show example images. Only fixations that fell on a person feature are shown, first fixations are marked as empty, following fixations as filled circles. Marker size is relative to dwell times. Panels (b, d, f) show example frames from three different videos shown in the experiment. Gaze samples are shown for the same two participants as in Panels (a, c, e).*

**
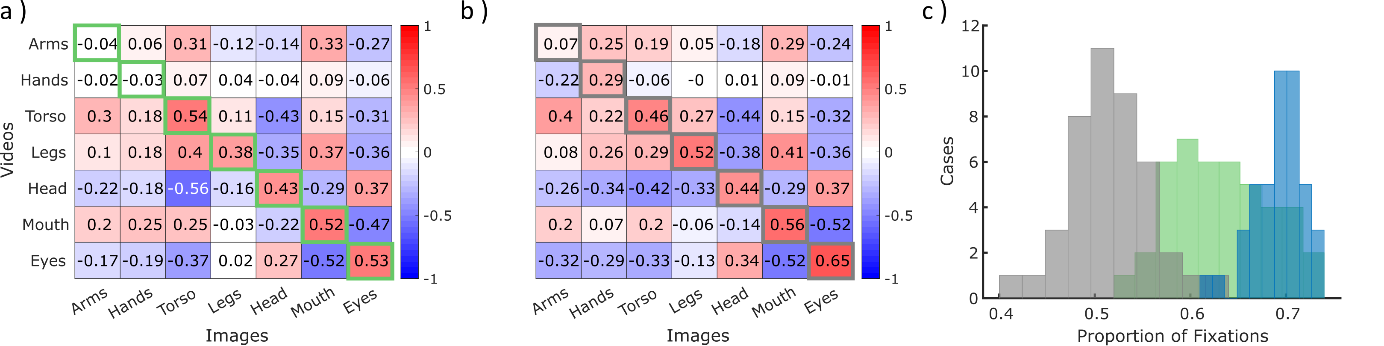
**

Figure S2 Consistencies and Correlations of images and videos

Panels (a, b) show the covariance patterns for proportions of video gaze samples and (a) proportions of first fixations and (b) proportional dwell time. Negative to positive correlations are indicated by color and saturation, as shown on the color bar to the right. (no significance tests). Panel (c) depicts the proportion of fixations falling on any person label across all fixations, separately for the proportion of first fixations (green), proportional dwell time (grey) and proportion of video gaze samples (blue). Neither fixation proportion in images correlated with the proportion of video gaze falling onto persons (both r ≤ .2, p > .19). This may be explained by the overall high proportion of person gaze for videos, with lower inter-individual variance (SD = 0.026) compared to the proportion of first fixations (SD = 0.053) or proportional dwell time (SD = 0.043).
